# Size-Specific Tree Mortality Varies with Neighbourhood Crowding and Disturbance in a Montane *Nothofagus* Forest

**DOI:** 10.1371/journal.pone.0026670

**Published:** 2011-10-26

**Authors:** Jennifer M. Hurst, Robert B. Allen, David A. Coomes, Richard P. Duncan

**Affiliations:** 1 Landcare Research, Lincoln, New Zealand; 2 Bio-Protection Research Centre, Lincoln University, Canterbury, New Zealand; 3 Forest Ecology and Conservation Group, Department of Plant Sciences, University of Cambridge, Cambridge, United Kingdom; Lakehead University, Canada

## Abstract

Tree mortality is a fundamental process governing forest dynamics, but understanding tree mortality patterns is challenging because large, long-term datasets are required. Describing size-specific mortality patterns can be especially difficult, due to few trees in larger size classes. We used permanent plot data from *Nothofagus solandri* var. *cliffortioides* (mountain beech) forest on the eastern slopes of the Southern Alps, New Zealand, where the fates of trees on 250 plots of 0.04 ha were followed, to examine: (1) patterns of size-specific mortality over three consecutive periods spanning 30 years, each characterised by different disturbance, and (2) the strength and direction of neighbourhood crowding effects on size-specific mortality rates. We found that the size-specific mortality function was U-shaped over the 30-year period as well as within two shorter periods characterised by small-scale pinhole beetle and windthrow disturbance. During a third period, characterised by earthquake disturbance, tree mortality was less size dependent. Small trees (<20 cm in diameter) were more likely to die, in all three periods, if surrounded by a high basal area of larger neighbours, suggesting that size-asymmetric competition for light was a major cause of mortality. In contrast, large trees (≥20 cm in diameter) were more likely to die in the first period if they had few neighbours, indicating that positive crowding effects were sometimes important for survival of large trees. Overall our results suggest that temporal variability in size-specific mortality patterns, and positive interactions between large trees, may sometimes need to be incorporated into models of forest dynamics.

## Introduction

Size-specific mortality rates of trees have a fundamental influence on the structure [1]–[3] and composition [4], [5] of forests, influence geographical range limits [6], determine forest carbon storage capacity [7], and can be sensitive to climatic change [8], [9]. A U-shaped size-specific tree mortality pattern is sometimes observed when measurements are made over large areas or long time frames [10]–[12]. Such a pattern is thought to be largely a consequence of asymmetric competition for light causing relatively high mortality of small trees and exogenous disturbance often causing relatively high mortality of large trees, while trees of intermediate size are less affected by either process [4], [13].

A wide range of abiotic and biotic factors can cause tree mortality and includes both random and deterministic events (e.g. [14]–[16]). Some factors may weaken trees while other's directly cause tree mortality [17]. Competition for light among neighbours has long been considered a key factor controlling tree death, particularly for small trees in a population [18]. Light competition is strongly size-asymmetric because light is directionally supplied from above and pre-empted by larger individuals, in contrast to competition for below-ground resources, which is usually assumed to be size-symmetric [19]. Shaded plants often have relatively slow growth and are more likely to die (e.g. [5], [20]–[23]). Because size-asymmetric competition is less important for taller trees, which are on average less shaded by neighbours, mortality rates should progressively decline with tree size if size-asymmetric competition is the dominant cause of tree mortality [24].

Exogenous disturbance is also a major cause of tree mortality [25], but its size dependence is hard to quantify because the many types of disturbance differ in their impacts. Strong windstorms commonly cause greater mortality among larger or taller trees [26]–[28], so windstorm damage is predicted to generate an upwardly rising tail leading to a U-shaped size-specific mortality curve. Indeed, for large trees, competition may be such an unimportant cause of mortality, relative to disturbance, that the loss of competing neighbouring canopy trees may even increase mortality of remaining trees because of increased susceptibility to disturbance (e.g. [29], [30]). Such a shift in the effects of neighbourhood crowding with increasing tree size, from competitive (i.e. negative neighbourhood crowding effects) to positive neighbourhood crowding effects, would be expected if a later life stage was particularly susceptible to certain stress [31], [32] or disturbance mechanisms [33].

A challenge for our study is to understand reasons for variability observed in the shape of size-specific mortality patterns (e.g. [34], [35]). One explanation for such variability is that over small spatial or temporal scales, either competition or disturbance may have a dominant influence on forest structure and dynamics [4], [10]. In addition, not all disturbances are size-discriminative and infrequent disturbance events such as landslides, hurricanes, tornados, volcanic eruption and earthquakes may kill trees across all size-classes (e.g. [36]). Depending on the nature of disturbance, size-specific mortality curves could change dramatically in form, from U-shaped to essentially size invariant, yet few studies have explored how the form of these curves, and the mechanisms behind them, change over time in the presence of different disturbance events (e.g. [37]).

This paper examines how size-specific mortality is affected by neighbourhood crowding and different types of disturbance, using a Bayesian framework to model individual tree mortality. We work with data collected over 30 years from tagged individuals on permanently marked plots within a single-species, mixed-aged forest. Our analyses allow us to evaluate how mortality varied in relation to risk factors for individual trees, in contrast to earlier characterizations of stand-level mortality rates [3]. *Nothofagus solandri* var. *cliffortioides* (Hook. f.) Poole (mountain beech) grows naturally on the eastern side of New Zealand's Southern Alps and forms monospecific stands over most of its range. It is a relatively light demanding species [38] so individuals growing with taller neighbours tend to have relatively slow growth rates [39]. The predominant disturbance agent within our forest varied over the study period. Snow and windstorms induced a pinhole beetle outbreak over the first 9 years, which mainly caused death of large trees [40], [41]. The forest was relatively stable over a second, 10-year period, when mortality chiefly resulted from small-scale windthrow and there was no evidence of major landscape-scale disturbance events. A third period was characterised by an earthquake that caused widespread tree mortality through landslides [36]. We model mortality processes in each of these three periods to unravel the ways in which disturbance and neighbourhood crowding affect size-specific mortality functions.

We hypothesised that U-shaped size-specific mortality patterns would be most apparent over long time intervals, which we addressed by examining mortality patterns over the entire 30-year period, and comparing this to patterns over each of the three periods characterised by different disturbance regimes. Specifically, we predicted that a U-shaped size-specific mortality pattern would be most evident where disturbances primarily impacted large trees (during the first and second periods) and less apparent in the third period where landslide disturbance tended to be size-indiscriminative [36]. By analysing individual-tree mortality over these three periods, with different disturbances, we attempted to evaluate the role of ‘regular’ versus ‘irregular’ drivers of size-specific mortality (e.g. [42]). We also hypothesised a shift from negative neighbourhood crowding effects (competitive) for small trees to positive neighbourhood crowding effects for large trees (particularly for the first two periods).

## Materials and Methods

### Study area and data collection

The 200-km^2^ study area (43°10′S, 171°35′E) centred on the Craigieburn Range, South Island, New Zealand, is mountainous with peaks over 2000 m in elevation and valley bottoms down to 600 m elevation. *Nothofagus solandri* var. *cliffortioides* is the dominant forest tree in this area, often forming monospecific stands, and living as long as 360 years but typically surviving <200 years [38]. We utilised plots sampling 9000 ha of forest that included stands up to the natural treeline at c. 1400 m elevation.

The underlying bedrock is formed of highly shattered, strongly indurated and intensely folded and faulted Triassic–Jurassic greywacke sandstones and mudstones [43]. These layers contain structural weaknesses and are prone to rock-avalanches triggered by earthquakes [44]. The soils are strongly leached, acidic and infertile (e.g. [45], [46]). At 914 m elevation on the eastern side of the study area, mean annual temperature is 8.0°C, dropping to 3.8°C at 1550 m elevation [47]. Mean annual precipitation on the eastern side of the study area is 1447 mm at 914 m elevation, rising to at least 1586 mm at 1550 m elevation [47]. An east–west rainfall gradient is present with the highest mean annual precipitation of 2500 mm occurring along the western edge of the study area [48].

Between 1970 and 1973, 250 permanently marked plots 20×20 m (0.04 ha) in size were established in mountain beech forest in the study area. Plots were located at 200-m intervals along transects, the origins of which were located randomly along watercourses, until alpine grassland was reached. Each plot was subdivided into 16 subplots of 5×5 m, within which the diameter at breast height (D; 135 cm above ground level) of all trees ≥3 cm D were recorded (see [49]). In 1974, plots were remeasured and all trees uniquely tagged at measurement height. Subsequent remeasurements identified dead tagged trees during the austral summers starting in 1976, 1978, 1980, 1983, 1985, 1987, 1993, 1999 and 2004.

**Figure 1:**
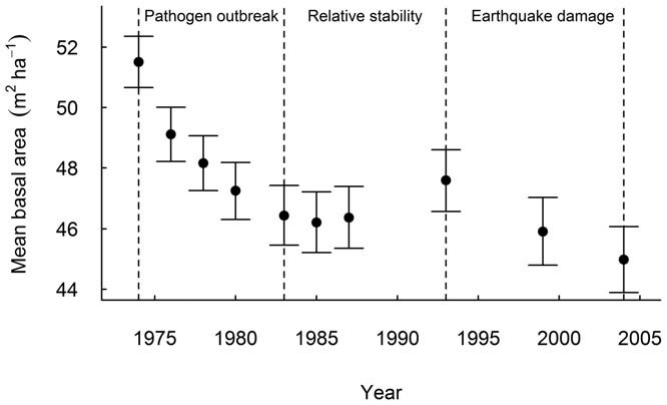
Basal area trend over the study period. Mean (± SEM) tree basal area (m^2^ ha^−1^) for study-area mountain beech forest from permanent plot measurements between 1970 and 2004. Dashed lines indicate the plot measurements used in this study to construct individual-based mortality models.

We utilise data from all measurements to characterise the forest disturbance history, but restrict our tree mortality analyses to the 1974, 1983, 1993 and 2004 plot measurements. Mean basal area declined from 51.5±0.85 m^2^ ha^−1^ in 1974, to 46.4±0.98 m^2^ ha^−1^ in 1983, due to a decade-long pinhole beetle outbreak (*Platypus* spp. and associated fungal pathogens) starting in 1970 and associated with woody debris created by unusually heavy snowfall and windstorms in 1968 and 1973; as well as ongoing windthrow (Fig. 1; [40], [41]). Overall mean basal area did not change during the 1983 and 1993 periods (Fig. 1), suggesting the forest was relatively stable and unaffected by major disturbance events, although windthrow and other causes of small-scale disturbance were observed [40]. An earthquake in 1994, with an epicentre 10 km north-west of the study area (Arthur's Pass earthquake, Mw 6.7), caused substantial damage to the forests, with mean basal area declining from 47.6±1.01 m^2^ ha^−1^ in 1993 to 45.0±1.09 m^2^ ha^−1^ by 2004 (Fig. 1). In a sub-catchment closest to the epicentre, containing 28 plots, earthquake-induced tree mortality was 24±5%, mostly as a result of widespread landslides [36]. Based on this information, we selected three periods with which to contrast size-dependent mortality (Fig. 1): two periods of forest decline (1974–1983 and 1993–2004) and one period of relative stability (1983–1993).

### Overall size-specific mortality pattern

**Figure 2:**
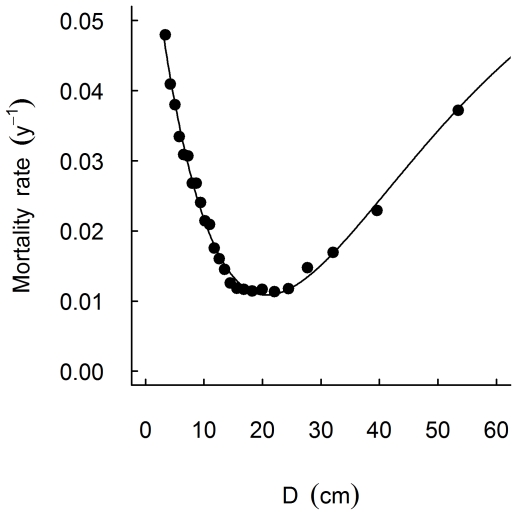
Size-specific mortality pattern over the 30-year study period, 1974–2004. Points represent the observed annual mortality rates, in size-class bins, plotted against the mean diameter (D) of stems in that size class. There were initially 19 515 trees alive in 1974, of which 9111 had died by 2004. Size-class bins each contained 1000 individuals, except for the largest size-class bin which contained 515 individuals. The line was fitted using the function: m = a+bD e^cD^, where a = 0.0680, b = −0.000752 and c = −0.00484.

To describe the way in which mortality varied with tree diameter, D, we started by fitting a model to the dataset for the entire 1974–2004 period. We first grouped trees into size-class bins, with each bin containing an equal number of trees. The annual mortality rate, m, for each bin was calculated and a function fitted to the binned data to describe the overall size-specific mortality pattern:
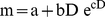
(1)where a, b and c are parameters. This functional form is highly flexible, allowing any initial decrease in mortality with increasing D for the smaller trees to be steeper than any increase in mortality with increasing D for larger trees [11]. This analysis revealed a U-shaped relationship between tree diameter and mortality, with a minimum mortality at a diameter of c. 20 cm (Fig. 2). On that basis we divided trees into small (3 cm≥D<20 cm) and large (D≥20 cm) classes (see also [2]) for fitting individual-based mortality models to test hypotheses regarding neighbourhood crowding effects for small v. large trees.

### Individual-based mortality models

The conventional approach to modelling tree mortality is to use logistic regression in a generalized linear modelling framework, where the survival probability of each tree is modelled as a linear combination of explanatory variables, such as tree diameter and competition indices [50], [51]. The response variable is a vector indicating whether or not a tree has died and the survival probability, S, of the ith tree, in the jth plot, over a census period of t years, can be expressed as a function of annual survival rate, s:
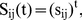
(2)where s is usually formulated using a logit link function to map the probability of survival S, which has range [0, 1], onto the numerical range (−∞, ∞ ), and k is a linear combination of explanatory variables that could affect tree survival:
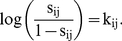
(3)Here, we build on this approach using a Bayesian framework to fit our models, in order to accommodate two complications arising from our dataset: first, we were interested in estimating the annual mortality rates of trees where the census intervals were not equal for the three periods (9, 10 and 11 years), and second, our sampling design is hierarchical (trees were sampled within plots). Assuming a constant annual probability of survival for each tree through a census period of length t, then the survival probability for tree i, in plot j, expressed as a function of its annual mortality rate, m_ij_, is:
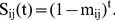
(4)Using the logit link function, the survival probability of the ith tree in the jth plot is:
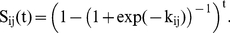
(5)


Our sampling design was nested because trees were measured in plots, so each tree is unlikely to represent an independent observation with regard to its probability of mortality. We dealt with this likely non-independence by including a random effect parameter, α, which took a unique value for each plot modelled as being drawn from a normal distribution with variance estimated from the data:
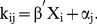
(6)


We also initially considered models without this random plot effect, but found that the plot effect improved model fit sufficiently to justify its inclusion.

Having split the dataset into the small- and large-tree subsets, we proceeded to model the influence of neighbours on mortality using two neighbourhood crowding indices. The ‘size-symmetric’ model related m_ij_ to the initial basal area of all neighbours within a 15×15 m square centred on the 5×5 m subplot within which a tree was recorded (BA_ij_ in m^2^ plot^−1^), while the ‘size-asymmetric’ model related m_ij_ to the initial basal area of larger-diameter neighbours within a 15×15 m square centred on the 5×5 m square within which a tree was recorded (BAL_ij_ in m^2^ plot^−1^). Using a subset of the data (750 random trees for which height was also measured) there was a strong positive relationship between D and individual height (Spearman's Rank Correlation 0.63, *P*<0.001) indicating that large-diameter trees are often tall trees, supporting the use of BAL as a proxy for potential shading effect on neighbours (e.g. [34], [52], [53]). In order to calculate these indices of crowding, only those trees growing in the central four 5×5 m subplots of each permanent 20×20 m plot could be used. For each census period, crowding indices were calculated using the census data from the beginning of the period (e.g. 1974 data were used for the 1974–1983 period).

To examine the potential for collinearity amongst explanatory variables, we calculated Variance Inflation Factors (VIF) for each possible combination of variables [54]. A conservative approach is to only fit combinations of variables that have VIFs<3 [54]. Our asymmetric (i.e. BAL) and symmetric (i.e. BA) neighbourhood crowding indices strongly covaried and typically resulted in VIF>3, so we did not include both indices in the same model. For other combinations of variables VIF was always <1.5, well below the level at which collinearity between variables is likely to be problematic [54].

Separate individual-based mortality analyses were conducted for the three census periods (1974–1983, 1983–1993, and 1993–2004) for small and large trees. Sample sizes ranged from 2876 to 3746 for small trees, and 1168 to 1257 for large trees. We first compared support for size-symmetric (BA) and size-asymmetric (BAL) models (both models included D as a main effect), for small and large trees to determine which of the crowding indices was more strongly associated with individual tree mortality (see next section for method of comparing model support). Second, for the best supported model we then included an interaction between D and the relevant neighbourhood crowding index (either BA or BAL) to develop a full individual-based mortality model. Finally, we used these models to assess the effect of explanatory variables and interactions. The sign of estimated parameter values for the two crowding indices indicated whether neighbourhood effects were competitive (i.e. positive parameter values) or positive (i.e. negative parameter values). Our full model containing the size-symmetric neighbourhood crowding index was:
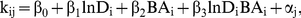
(7)while the full model containing the size-asymmetric neighbourhood crowding index was:
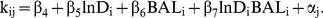
(8)


### Model fitting and comparison of alternative models

The first step in fitting our Bayesian models was to define, for each model, the likelihood function. During each census period there were j = 1 to K plots, with each plot having i = 1 to N_j_ trees alive at the first census. At the end of each census period, tree i in plot j was assigned a value of 1 if it died in that census period (d_ij_ = 1) or zero if it remained alive (d_ij_ = 0). Given the probability that tree i in plot j survived to time t is S_ij_(t), and the probability that it died in the same period is 1−S_ij_(t), the likelihood function for our sample of trees is:
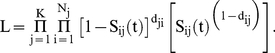
(9)


We estimated the parameters in our models using this likelihood function in a Bayesian model framework using *OpenBugs* v2.10 [55] called from the *BRugs* package from R v. 2.7.0 [56]. These methods were chosen because they allow for simple and efficient estimation of parameters and their confidence intervals, and allowing uncertainty in parameter estimates to be directly propagated into predictions. To improve model convergence and computation time, the explanatory variables were standardised by subtracting their mean and dividing by two standard deviations (e.g., [57]; see Table S2).

The next step in fitting our Bayesian models was to give all parameters starting values and a prior distribution [57]. In our case these were non-informative, to allow the data to drive parameter estimation. The fixed-effect parameters were assigned normal prior distributions with mean 0 and standard deviation 100. The variance term for the plot random effect was given a broad uniform prior on the standard deviation [57].

To run the models we performed three MCMC (Markov Chain Monte Carlo) simulations with different starting values, to provide confidence in the model results. For all runs of the models, a burn-in phase of 100 000 iterations was identified as suitable through visual examination of the chain traces, to ensure each model had converged. We continued each MCMC run for a further 100 000 iterations and used the last 50 000 iterations of all three runs (i.e. a sample of 150 000 in total) to obtain posterior distributions for each parameter. From these we derived mean values and 95% credible intervals. For each model, we checked convergence for each parameter using the potential scale reduction factor 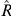 (at convergence 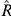 = 1; [57]).

Finally, we used the Deviance Information Criterion (DIC) to compare the relative fit of the size-symmetric and size-asymmetric models. DIC balances model fit with complexity, and is given by 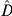
+2pD, where 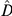 is a point estimate of the deviance at the posterior mean of the parameters, and pD is the ‘effective number of parameters’ [57], [58]. Models with lower DIC values indicate a better fit to the data, where differences ≥5 are regarded as substantial evidence, and differences ≥10 are regarded as very strong evidence, in favour of the model with the lowest DIC.

### Relationships between explanatory variables and mortality

From the best-supported models for small and large trees, over each census period, we used the sampled posterior distribution for each parameter to examine relationships between significant explanatory variables (e.g. D, BA, BAL) and mortality rate. For example, for each value of BAL across the range in our original dataset we sampled 20 000 times from the parameter estimate posterior distributions of the appropriate model, calculated the predicted mortality rate and graphed the mean and 95% credible interval for each value of BAL. We used a similar procedure to plot significant predicted mean relationships between D and mortality rate, as well as predicted mortality at low (5% quantile of values in our sample) and high (95% quantile of values in our sample) levels of neighbourhood crowding (using BAL for small trees and BA for large trees) when significant.

### Assessing model fit

We took several approaches to appraise the adequacy of the final models. For small and large trees, over each census period, the final models were used to estimate a mortality probability for each tree, using the mean values of the posterior distribution of each parameter estimate. Trees were grouped by mortality probability and the proportion of individuals in each group that died during each census period determined (e.g. [26], [35]). In a well-fitting model, the proportion of individuals in each probability group that died will be roughly equal to the midpoint of the probability interval. To assess the discriminatory power of all models we also calculated the AUC (area under the receiver operating characteristic curve), using the ROCR Package in R 2.7.0 [59]. AUC values>0.8 indicate a model has excellent discriminatory power and values>0.7 indicate good discriminatory power [60].

## Results

### Overall mortality rates and size dependence

**Figure 3:**
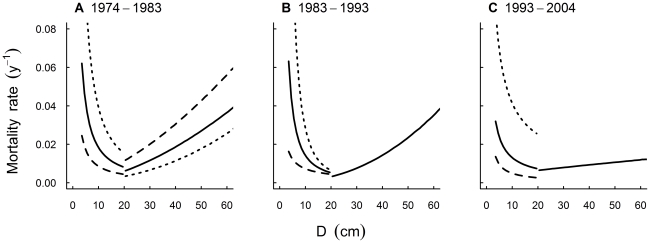
Temporal variation in size-specific mortality patterns. Size-specific mortality patterns for the three census periods, (**A**) 1974–1983, (**B**) 1983–1993 and (**C**) 1993–2004; based on separate analyses for small (D<20 cm) and large (D≥20 cm) trees. Solid lines show the significant modelled relationships between mortality and D determined from a simulation drawing on the posterior distribution of parameter estimates. For small trees (**A**–**C**), and large trees (**A**), the dashed and dotted lines show the significant relationships with neighbourhood crowding (i.e. basal area of larger neighbours (BAL) for small trees, basal area of all neighbours (BA) for large trees), representing low (dashed) and high (dotted) neighbourhood crowding (the 5% and 95% quantiles of BAL/BA for small and large trees) respectively.

The overall mean annual mortality rates were 0.022, 0.019, and 0.018 through the 1974–1983, 1983–1993, and 1993–2004 periods respectively. The size-specific mortality function was U-shaped over the 30-year study period with observed mortality rates highest in the smallest sized trees (e.g. maximum rate c. 0.05 for trees c. 3.5 cm D), least in intermediate sized trees (minimum rate c. 0.01 for trees c. 20 cm D) and then steadily increased for the largest trees (maximum rate c. 0.04; Fig. 2). Notably the decline in mortality with increasing tree size for small trees was much steeper than the increase in mortality with size for large stems. Similar patterns were observed over the 1974–1983 and 1983–1993 periods, but not over the 1993–2004 period (Fig. 3).

**Figure 4:**
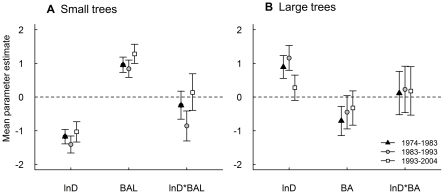
Mean parameter estimates for full individual-based mortality models. Parameter estimates are shown for (**A**) small (D<20 cm) and (**B**) large (D≥20 cm) trees. Each group of three points, for each parameter, represents estimates for the 1974–1983 (black triangle), 1983–1993 (grey circle) and 1993–2004 (white square) census periods. Bars show the 95% credible interval for the parameter estimates. D = diameter (mm), BAL = basal area of larger neighbours (m^2^ plot^−1^), BA = basal area of all neighbours (m^2^ plot^−1^).

For small trees, the estimated mean parameter values for D were consistently negative (Fig. 4; Table S1) over all three periods, indicating a decline in mortality with tree size (Fig. 3). We noted an anomaly, however, where observed mortality rates (compared with modelled rates) during the 1993–2004 period were much lower for the very smallest trees (e.g. <6 cm D; Fig. 3); further analyses showed this pattern was driven by trees in stands with low mean D (<6 cm).

For large trees, individual-based models reflected the variable size-dependence of mortality we observed between periods (Fig. 3). Over the 1974–1983 and 1983–1993 periods mortality increased with tree size, as shown by the positive mean parameter estimates for D (Fig. 4; Table S1). Over the 1993–2004 period, as we hypothesised, mortality was size-independent – as the 95% credible intervals for the mean parameter estimates for D intersected zero (Fig. 4).

### Effects of neighbourhood

**Figure 5:**
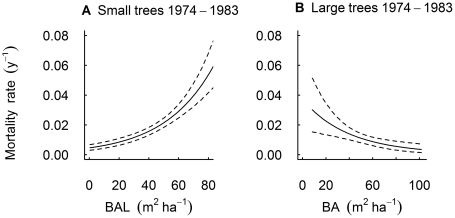
Predicted mortality against neighbourhood crowding variables. Predicted effect of (**A**) basal area of larger neighbours (BAL, m^2^ ha^−1^) on mortality for small (D<20 cm) trees, and (**B**) basal area (BA, m^2^ ha^−1^) on mortality for large (D≥20 cm) trees over the 1974–1983 census period. The solid line shows predicted relationships as determined from simulations drawing on the posterior distribution of parameter estimates. Dashed lines show the 95% credible intervals of the relationship.

For small trees, during all three periods, the size-asymmetric model fitted the data better than the size-symmetric model, as indicated by lower DIC values, with the difference between these models >20 in each period (Table 1). The estimated mean parameter values for the size-asymmetric terms were positive for all periods (Fig. 4), implying higher rates of mortality among trees with more large neighbours (higher BAL; Fig. 5a). This is consistent with our hypothesis that crowding has negative (i.e. competitive) neighbourhood effects and contributes to the death of small trees. Negative values for an interaction term between D and BAL in the full model for the 1983–1993 period of relative stability (Fig. 4) indicated that the influence of BAL on mortality declined with increasing D (Fig. 3).

**Table 1:** Model selection statistics for alternative mortality models.

Tree size and census period	Model	ΔDIC	AUC
**Small trees (D<20 cm)**			
1974–1983	size-asymmetric model	0	0.791
	size-symmetric model	56	0.784
	full size-asymmetric model	−1	0.793
1983–1993	size-asymmetric model	0	0.834
	size-symmetric model	27	0.828
	full size-asymmetric model	−12	0.838
1993–2004	size-asymmetric model	0	0.836
	size-symmetric model	26	0.832
	full size-asymmetric model	1	0.836
**Large trees (D≥20 cm)**			
1974–1983	size-asymmetric model	4	0.878
	size-symmetric model	0	0.865
	full size-symmetric model	1	0.866
1983–1993	size-asymmetric model	4	0.863
	size-symmetric model	0	0.866
	full size-symmetric model	3	0.861
1993–2004	size-asymmetric model	0	0.909
	size-symmetric model	2	0.907
	full size-symmetric model	3	0.907

Models describe mortality processes for small (D<20 cm) and large (D≥20 cm) trees during three census periods. Models were fitted in a Bayesian framework using MCMC (Markov chain Monte Carlo) simulations. DIC (Deviance Information Criterion) was used to identify the best-fitting model [51], the model with the lowest DIC having strongest support. ΔDIC is the difference in DIC between the best-fitting ‘main effects’ model (either the size-symmetric or size-asymmetric model) and the two other alternatives, with the best initial model having ΔDIC = 0. Negative ΔDIC values for full models (which include interactions) indicate that the incorporation of the interaction improved the model. AUC provides a measure of overall accuracy of the model at all probability thresholds [53].

For large trees, there was evidence that the size-symmetric models fitted the data better than size-asymmetric models in the 1974–1983 and 1983–1993 periods (Table 1), whereas there were few grounds to prefer either model in the 1993–2004 period (i.e. ΔDIC = 2). Over the 1974–1983 period the mean parameter value for the size-asymmetric term (BA) was significantly negative (Fig. 4) implying higher rates of mortality among trees with lower neighbouring basal area (Fig. 5b). For this period, the pattern is consistent with our hypothesis that positive neighbourhood crowding effects can be important for the survival of large trees. Mean parameter estimates for BA were also negative in the 1983–1993 and 1993–2004 periods (Fig. 4), although in neither case were these significant. We proceeded to fit the full size-symmetric model for all three periods but in no cases did this model receive greater support than the simpler model (Table 1), and an interaction term between BA and D was not significant for any period (Fig. 4).

### Assessing model fit

The discriminatory power of the models to correctly identify living and dead trees, as measured by the AUC, ranged from good (0.7>AUC<0.8) to high (AUC>0.8) [54] for all models (Table 1), and in general the AUC value results were congruent with the best supported models as measured by DIC (Table 1). A close correspondence between the observed proportion of trees that died against predicted mortality probability was observed, but discrepancies from the observed values did occur, typically as underestimates of mortality and especially when the predicted number of trees in a probability category was low (see Figure S1). It is also notable that the model describing large tree mortality in the 1993–2004 period resulted in high discriminatory power for predicting mortality despite the fact that none of the included variables were significant (Table 1; Fig. 4). The distribution of fitted plot-level effects, α_j_, had greater variance over this period, when compared with the earlier periods (e.g. 1.94±0.0 for 1993–2004 cf. 0.86±0.0 for 1974–1983 and 0.79±0.0 for 1983–1993), as a consequence of the strongly clustered mortality that resulted from earthquake-induced landslides.

## Discussion

### Size-specific mortality patterns and the influence of disturbance

We found strong support for U-shaped size-specific mortality functions in the mountain beech forest, particularly, as hypothesised, over the longest time interval. Determining precise mortality estimates for large trees can be problematic, as they often comprise a small proportion of a tree population. Large tree mortality rates, however, have a dominant influence on forest structure and have a key influence on the results of predictive models of forest dynamics. Our 30-year dataset for mountain beech, a relatively light demanding structural dominant, was sufficient to characterise a pattern of increasing mortality with size for larger trees. Large trees, rather than small trees, may be more predisposed to the range of small-scale disturbance factors that operate in these forests. Windthrow, in particular, is a common feature in mountain beech forests that influences large trees and was likely to occur over our periods of both forest decline and stability [40]. Higher mortality rates in large trees may also be attributed to declining vigour and senescence (e.g. [53]). Explanations for declining vigour include hydraulic limitation (e.g. [61]), an increasing ratio of sapwood volume to leaf area (e.g. [62]), and immobilization of nutrients (e.g. [63]).

The U-shaped mortality pattern we observed over the 30-year period broke down, as hypothesised, during the 1993–2004 period characterised by earthquake disturbance (Fig. 3), which was less size-discriminatory than the disturbances predominating during other periods. Major landscape-scale disturbances can also lead to structural changes in a forest that subsequently influence the form of population-level size-specific mortality patterns. For example, the anomaly we observed during the 1993–2004 period for small trees, of very low mortality in the smallest size classes, appeared to be driven by stands with low mean *D* (<6 cm). Stands dominated by such small trees became more frequent in that period as a consequence of a regeneration lag following the earlier *Platypus* spp. outbreak. Because we sampled only those stems with diameter ≥3 cm, it is likely that competition and self-thinning in such stands caused high mortality of individuals below our tagging threshold. Few studies adequately capture variation in size-specific mortality patterns as a result of different landscape-scale disturbance agents. Woods [27] found that a storm increased mortality rates of intermediate to large trees in temperate old-growth hemlock–hardwood forest. In tropical rainforest, mortality became less size-dependent when a severe drought caused death of trees across all size-classes [64]; however, in another study, drought resulted in higher mortality rates among larger trees, while fire resulted in high mortality rates among small trees [37]. Thus, our study contributes to a view that, through time, disturbance can result in different size-specific mortality functions. That the form of size-specific mortality functions were idiosyncratic reflects that tree mortality is a complex process [17], [53], and depended on context, is consistent with conclusions around other ecosystem properties (e.g. [65]).

### Effects of neighbourhood crowding

Our individual-level mortality models allowed us to show that neighbourhood effects on mortality varied through time, a pattern not previously exposed using simple stand-level approaches (e.g. as adopted by [3]). Our study also found that the net effect of neighbours on mortality shifted from negative crowding effects for small trees to positive or no crowding effects for large trees. While a shift in neighbourhood effects with plant size or life-stage is certainly not without precedent (e.g. [66]) our study demonstrates that neighbours can sometimes have very different effects on small- and large-tree performance. Small trees are likely to have greater susceptibility to competition-driven mortality (cf. large trees), as we know negative neighbourhood crowding (asymmetric competition for light) was a key determinant of growth rate in small *Nothofagus solandri* var. *cliffortioides* trees [3]. However, small tree mortality is not always controlled by competition from larger neighbours. Light competition is less important for small trees of shade-tolerant species [21], [67], while certain types of disturbance (e.g., earthquakes) can drive mortality patterns that are unrelated to neighbourhood crowding [36]. Small trees run a greater risk of being crushed and killed by litterfall (e.g. falling stems and branches) from large neighbours [68], [69]. Larger trees, however, tend to be more susceptible to disturbance. We expected that mortality of large trees would be positively influenced by neighbours in periods dominated by small-scale disturbance. The precise mechanisms behind positive neighbourhood interactions sometimes being found for large *Nothofagus solandri* var. *cliffortioides* trees remain speculative. We interpret the important effect of neighbourhood crowding during the 1974–1983 period as a direct reflection of the disturbance processes that predominated during that period. For example, *Nothofagus* stands with low basal area caused by previous disturbances, such as snowfall damage, often harbour large quantities of woody debris which serves as a breeding ground for the disease-causing pinhole beetle [40]. These beetles are known to increase mortality among residual trees, and this may be an example of a positive disturbance interaction (sensu [33]). Pests and pathogens are generally an important cause of ongoing tree death [17], [70], and their impacts can be related to climate variation (e.g. [9]). Crowded stands may also provide supportive or sheltering effects on large trees, which may lessen any impact of disturbance agents such as wind (e.g. a facilitative effect). This contrasts with some previous studies which have shown facilitative effects to generally only benefit younger or smaller plants (e.g. nurse plants facilitate survival of smaller seedlings in semi-arid systems; [71]). While the overall size-specific mortality pattern observed in the 1974–1983 and 1983–1993 periods were remarkably similar, the different results with respect to neighbourhood crowding suggest that various disturbance types differed in the degree to which they caused contagion in tree mortality. It was however unsurprising that positive neighbourhood crowding effects were not observed during the 1993–2004 period, because of the indiscriminatory nature of earthquake induced mortality [36].

### Conclusion

Determining tree mortality and turnover rates in forests is essential to understand pest and pathogen impacts (e.g. [72]), the effects of climatic change on forests [8] and for developing simulation models of forest dynamics [73]. In addition, simulation models appear very sensitive to the form of the mortality function employed (e.g. SORTIE [74]). Our results support a view that these models may need to include variation in mortality functions through time to adequately represent dynamic forest systems.

## Supporting Information

Figure S1 **Goodness of fit of the full individual-based models.** Goodness of fit graphs (see Table 1) for small (D<20 cm) and large (D≥20 cm) trees, for each of three census periods: 1974–1983, 1983–1993 and 1993–2004. Points represent the observed proportion of trees that died as a function of predicted mortality probability, and numbers above points indicate the number of observations in each probability class. Diagonal lines represent a 1∶1 relationship between observed and predicted mortality.
(TIF)

Table S1 Parameter estimates for full individual-based models. Mean (±SD) lower 95% and upper 95% of posterior distribution of sampled parameter estimates for individual-based mortality models for small (D<20 cm) and large (D≥20 cm) trees. Note that input variables were centred and standardised before inclusion in individual-based models (see Table S2b for mean values for each variable).
(DOCX)

Table S2 Mean (±SD) of each variable in the raw data, across three census periods, for small (D<20 cm) and large (D≥20 cm) trees.
(DOCX)
