# Supplementary figures and images for: Size-Specific Tree Mortality Varies with Neighbourhood Crowding and Disturbance in a Montane Nothofagus Forest
Source: PLoS One. 2011 Oct 26;6(10):e26670. doi: 10.1371/journal.pone.0026670 (PMC3202550; doi:10.1371/journal.pone.0026670)

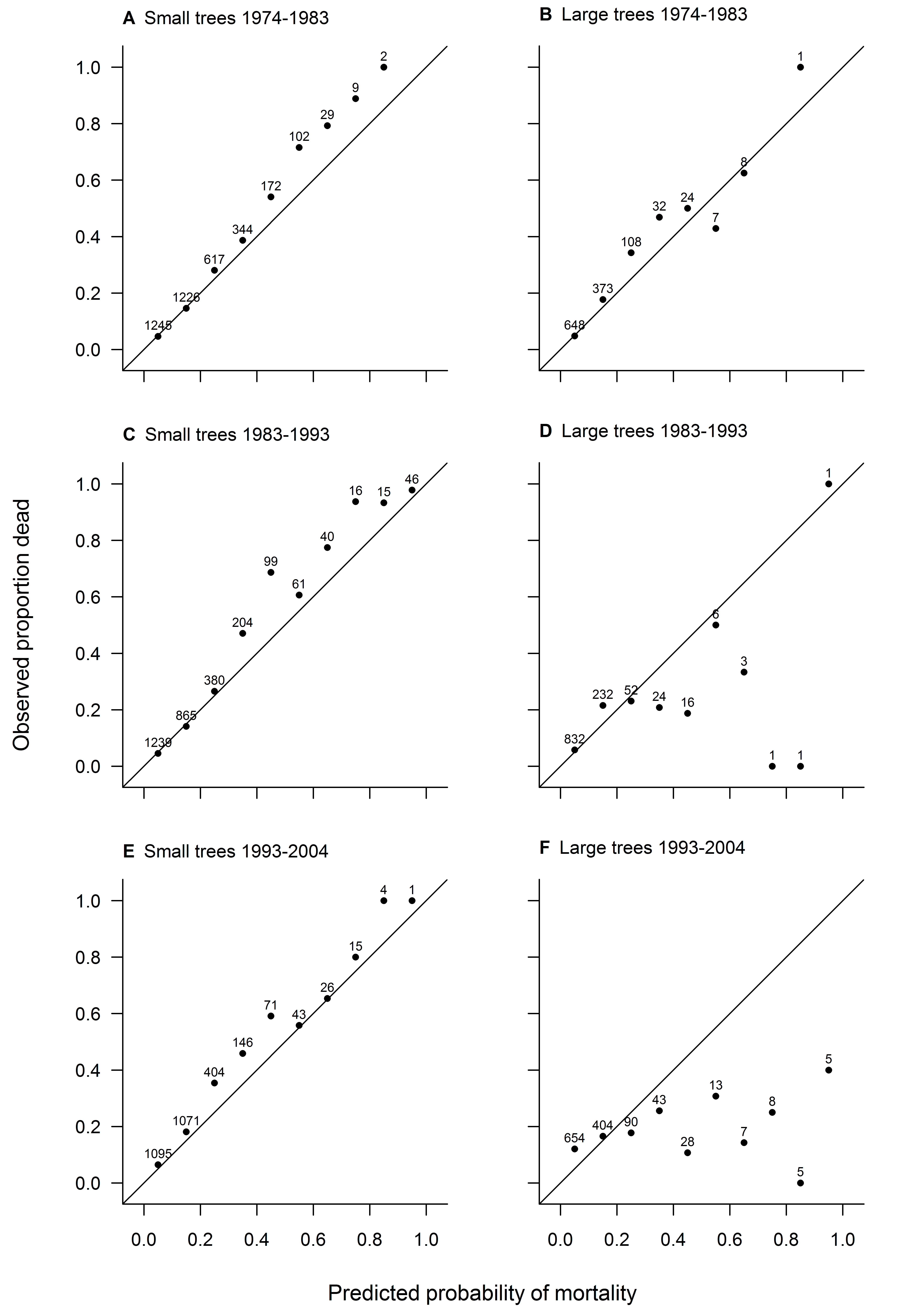

Supplement: Figure S1 — Goodness of fit of the full individual-based models. Goodness of fit graphs (see Table 1) for small (D<20 cm) and large (D≥20 cm) trees, for each of three census periods: 1974–1983, 1983–1993 and 1993–2004. Points represent the observed proportion of trees that died as a function of predicted mortality probability, and numbers above points indicate the number of observations in each probability class. Diagonal lines represent a 1∶1 relationship between observed and predicted mortality. (TIF) [file pone.0026670.s001.tif]
