# Supplementary material for: Size-Specific Tree Mortality Varies with Neighbourhood Crowding and Disturbance in a Montane Nothofagus Forest
Source: PLoS One. 2011 Oct 26;6(10):e26670. doi: 10.1371/journal.pone.0026670 (PMC3202550; doi:10.1371/journal.pone.0026670)
Supplement: Table S1 — Parameter estimates for full individual-based models. Mean (±SD) lower 95% and upper 95% of posterior distribution of sampled parameter estimates for individual-based mortality models for small (D<20 cm) and large (D≥20 cm) trees. Note that input variables were centred and standardised before inclusion in individual-based models (see Table S2b for mean values for each variable). (DOCX) [file pone.0026670.s002.docx]

| **Size class** | **Census period** | **Parameter** | **Mean** | **SD** | **Lower 95%** | **Upper 95%** |
| --- | --- | --- | --- | --- | --- | --- |
| Small trees | 1974–1983 | Intercept | –3.921 | 0.078 | –4.076 | –3.771 |
|  |  | lnD | –1.174 | 0.109 | –1.390 | –0.964 |
|  |  | BAL | 0.954 | 0.116 | 0.731 | 1.186 |
|  |  | lnD*BAL | –0.246 | 0.214 | –0.657 | 0.175 |
| Small trees | 1983–1993 | Intercept | –4.246 | 0.090 | –4.428 | –4.072 |
|  |  | lnD | –1.407 | 0.129 | –1.662 | –1.156 |
|  |  | BAL | 0.839 | 0.132 | 0.582 | 1.098 |
|  |  | lnD*BAL | –0.856 | 0.228 | –1.304 | –0.413 |
| Small trees | 1993–2004 | Intercept | –4.204 | 0.112 | –4.431 | –3.992 |
|  |  | lnD | –1.032 | 0.154 | –1.336 | –0.732 |
|  |  | BAL | 1.281 | 0.145 | 0.998 | 1.569 |
|  |  | lnD*BAL | 0.138 | 0.278 | –0.395 | 0.692 |
| Large trees | 1974–1983 | Intercept | –4.529 | 0.147 | –4.843 | –4.262 |
|  |  | lnD | 0.893 | 0.175 | 0.554 | 1.234 |
|  |  | BA | –0.706 | 0.221 | –1.145 | –0.278 |
|  |  | lnD*BA | 0.115 | 0.330 | –0.523 | 0.763 |
| Large trees | 1983–1993 | Intercept | –5.017 | 0.179 | –5.390 | –4.680 |
|  |  | lnD | 1.158 | 0.186 | 0.795 | 1.531 |
|  |  | BA | –0.448 | 0.253 | –0.946 | 0.040 |
|  |  | lnD*BA | 0.227 | 0.355 | –0.464 | 0.917 |
| Large trees | 1993–2004 | Intercept | –4.874 | 0.171 | –5.224 | –4.558 |
|  |  | lnD | 0.278 | 0.191 | –0.100 | 0.652 |
|  |  | BA | –0.323 | 0.260 | –0.836 | 0.182 |
|  |  | lnD*BA | 0.178 | 0.365 | –0.537 | 0.904 |
