# Supplementary material for: Size-Specific Tree Mortality Varies with Neighbourhood Crowding and Disturbance in a Montane Nothofagus Forest
Source: PLoS One. 2011 Oct 26;6(10):e26670. doi: 10.1371/journal.pone.0026670 (PMC3202550; doi:10.1371/journal.pone.0026670)
Supplement: Table S2 — Mean (±SD) of each variable in the raw data, across three census periods, for small (D<20 cm) and large (D≥20 cm) trees. (DOCX) [file pone.0026670.s003.docx]

| **Size class** | **Census period** | **D** | **BAL** | **BA** |
| --- | --- | --- | --- | --- |
| Small trees | 1974–1983 | 105.6 (45.7) | 1.055 (0.339) |  |
|  | 1983–1993 | 110.9 (46.1) | 0.993 (0.371) |  |
|  | 1993–2004 | 101.1 (52.0) | 0.953 (0.404) |  |
| Large trees | 1974–1983 | 298.0 (89.1) |  | 1.158 (0.341) |
|  | 1983–1993 | 293.5 (86.0) |  | 1.095 (0.374) |
|  | 1993–2004 | 294.0 (84.3) |  | 1.136 (0.394) |
